# Quality of life among cancer patients at Queen Elizabeth and Kamuzu Central Hospitals in Malawi: a cross-sectional double-center study

**DOI:** 10.4314/ahs.v22i3.24

**Published:** 2022-09

**Authors:** Jonathan Chiwanda Banda, Maganizo B Chagomerana, Michael Udedi, Adamson Sinjani Muula

**Affiliations:** 1 Department of Public Health, Kamuzu University of Health Sciences; 2 The Africa Center of Excellence in Public Health and Herbal Medicine, Kamuzu University of Health Sciences; 3 Non-Communicable Disease Unit, Clinical Services Department, Ministry of Health, Malawi; 4 University of North Carolina Project, Lilongwe, Malawi; 5 Department of Epidemiology, University of North Carolina-Chapel Hill, 135 Dauer Dr, Chapel Hill, North Carolina, 27599-7435, United States; 6 Department of Medicine, University of North Carolina, Chapel Hill, North Carolina, USA

**Keywords:** cancer patients, quality of life

## Abstract

**Introduction:**

Many cancer patients experience psychosocial challenges that affect quality of life during the trajectory of their disease process. We aimed at estimating quality of life among cancer patients at two major tertiary hospitals in Malawi.

**Methods:**

The study was conducted among 398 cancer patients using semi-structured questionnaire. Quality of life was measured using EQ-5D-3L instrument.

**Results:**

Mean age was 45 years ± 12.77. Pain (44%) was the most prevalent problem experienced by cancer patients. About 23% had worst imaginable health status on the subjective visual analogues scale. Attending cancer services at QECH (AOR= 0.29, 95% CI: 0.17–0.54, p<0.001) and having normal weight (AOR=0.25, 95% CI: 0.08–0.74, p = 0.012), were associated with improved quality of life. A history of ever taken alcohol (AOR= 2.36, 95% CI: 1.02–5.44, p = 0.045) and multiple disease comorbidities (AOR= 3.78, 95% CI: 1.08–13.12, p = 0.037) were associated with poor quality of life.

**Conclusion:**

Loss of earning, pain, marital strife, sexual dysfunction, were among the common psychosocial challenges experienced. History of ever taken alcohol and multiple comorbidities were associated with poor quality of life. There is need to integrate psychosocial solutions for cancer patients to improve their quality of life and outcomes.

## Introduction

Cancer incidence and mortality are on the increase and remain one of the major public health problems worldwide^1^. According to GLOBOCAN 2020 report, an estimated 19.3 million new cancer cases and 10.0 million cancer deaths occurred in 2020 2. Furthermore, over 36 million people were living with various forms of cancer and the burden disproportionately affected Low- and Middle-Income Countries (LMICs) which contributed 70% of cancer deaths in 2020 3. In Malawi, cancers contributed to 16% of Disability-Adjusted Life Years (DALYs) due to Non-Communicable Diseases (NCDs) in 2015 4, 5. Cancer survival in Malawi was equally poor with median survival time of about 9 months and only 6% of patients surviving for 5 years or more in 2014 6. The top five common cancers in both genders included: cervical cancer (23.1%), esophageal (9.8%), Kaposi sarcoma (9.4%), breast cancer (8.3%) and non-Hodgkin's lymphoma (6.5%) 7.

People living with cancer experience several challenges that affect their quality of life (QoL) 8, 9. The World Health organization (WHO) defines QoL as individual's perception of their position in life in the context of the culture and value systems in which they live and in relation to their goals, expectations, standards and concern 10. Therefore, quality of life in cancer is a dynamic, multidimensional concept, referring to all life aspects and needs of the patient, continuously assessing balancing processes between the real and ideal situation at a given time 11. The factors affecting QoL among cancer patients are not only physiological in nature, but also psychological, arising from patient reaction to results of diagnostic tests, the stages of sadness, grief and anger 8.

Although several QoL instruments have been validated and used in different settings, most have reported low scores among cancer patients. A study in India reported 82% of the patients had low scores of QoL which were contributed by pain (73%), depression (54%), and physical deterioration (86%) among other indicators 12. In Poland, 87.5% of the patients had low scores which were attributable to severe problems of self-care (81%), and depression (63%) 11. Similarly in Iran, improved QoL was associated with improved income, higher educational status (p<0.05) 8. A similar study in Malaysia had also reported that 70% of the participants were depressed while 93% had anxiety 13. In Ethiopia, QoL was reduced with advanced cancer disease, ageing and low socioeconomic status 14. While in Kenya, marriage and education were associated with improved QoL 15. A multi-center study in South Africa and Uganda has shown that patients receiving palliative care exhibited significantly poorer QoL on function subscale followed by well-being, symptoms, transcendent and interpersonal subscales compared to similar populations in the High-Income Countries (HICs)^16^. Similarly in Malawi, few studies done on patients with various chronic illnesses under palliative care had shown poor quality of life 17, 18.

The measurement of QoL and follow-up may provide important information to ascertain acceptance, adaptation of cancer disease, treatment and could also represent end-line evaluation of healthcare interventions 9, 19.A rapid review of quality of life studies in Malawi indicated fourteen studies and only two of those focused on palliative care targeting chronic conditions including cancer^17^, ^18^. Again, only two other studies from that review had used EuroQol-5 Dimensions-3Levels (EQ-5D-3L) tool for evaluating patients' quality of life in Malawi and these included a validation study by Chokotho et al and a health-related quality of life of inpatients and outpatients treated for tuberculosis in rural Malawi by Jo et al^19^, ^20^. Lack of studies in Malawi evaluating QoL among cancer patients limits the possibility of ascertaining type of psychosocial problems experienced by these particular set of patients and how to approach them especially in resource limited settings 19. Therefore, the current study was aimed at estimating quality of life and the psychosocial experiences among cancer patients attending oncology services at Queen Elizabeth (QECH) and Kamuzu Central Hospitals (KCH), the two main referral hospitals in Malawi. The findings could support development of interventions to guide in effective management of psychosocial challenges and consequently helping empowering patients over their illnesses and treatment and possibly improve their QOL 12.

## Methods

### Study setting and design

We conducted a cross-sectional study at two main referral hospitals of QECH and KCH between January and March, 2021. Basing on monthly clinic data, these two facilities attend to majority of the cancer patients in the country with an average of 3,000 and 2,400 patients reviewed annually at QECH and KCH respectively. The study took place at oncology clinics as specialized centers where all newly diagnosed cancers are referred for further management. In this case we minimized selection bias of study participants. By the time of the conduct of the study, there were two oncologists, four non-specialist medical doctors and 13 nurses at QECH. On other hand at KCH, there was one oncologist, four non-specialist medical doctors and 26 nurses. In both sites, there were no psychosocial counsellors working at study sites and that standard practice was that patients suspected of psychosocial problems would be referred to psychiatric clinics which were situated away from cancer clinic premises.

### Sampling size and sampling technique

This study was embedded in a larger cancer comorbidity prevalence study whose sample size was estimated basing on the Cochran's formula using estimated prevalence of 26% of single count chronic disease comorbidity among cancer patients,5% precision level and 95% confidence level (Z= 1.96) 21. We recruited 398 participants for the study. We used simple random sampling approach using consecutive numbers in recruiting participants aged above 18 years of age attending to adult oncology clinics.

### Ethical Review and Approvals

The College of Medicine Research and Ethics Committee (COMREC) approved the study (P.07/20/3085). Approval l tters for conducting the study in the respective sites were obtained from Hospital Directors. Patient identity numbers at the clinics were used as codes to replace actual patient names to maintain confidentiality.

### Data collection and data management

Data were collected through face-to-face interviews using semi-structured questionnaire on socio-demographic and psychosocial factors. Quality of life was measured using EQ-5D-3L tool which had been previously validated in Malawi 19. This instrument was commissioned by the EuroQOL group in 2012 and it is a preference-based measure of health status 19. The instrument has two sections, the first part has 5 dimensions, namely: mobility; self-care; usual activities; pain/discomfort; and anxiety/depression. Each dimension has 3 levels: no problems, some problems, and extreme problems, with scores of 1, 2, and 3 representing each level, respectively. The respondents were asked to choose one level for each of the 5 dimensions that best described their own health state on the day of the interview^19^. The second section has a visual analogue scale (VAS), whereby patients would self-rate their health state on a scale of 0 to 100, with 0 and 100 as the worst and best imaginable health states, respectively^11^,^19^. The collected data was uploaded into Open Data Kit (ODK) on android tablets to minimize data collection errors and also reduce missing data. Data validations and checks were programmed to ensure that much of data capture errors are solved at the data collection point. All data collected on the tablets were being sent to a secured server and routine data quality checks were ran on the server to identify any data inconsistencies and discrepancies which were then sent to the data collection teams for resolutions which later applied to the server. Data were downloaded from the server as a CSV dataset which was imported into Stata for further data preparations and data analysis.

### Study variables

#### Outcome variable

The study considered quality of life as the primary outcome while psychosocial factors were the secondary outcome.

#### Independent variables

The study had the following explanatory variables (1) sociodemographic characteristics such as; sex, age, marital status, area of residence, education level, occupation, socioeconomic status, (2) behavioral risk factors such as smoking and alcohol (3) cancer diagnosis as it appears in the patient files and the date of diagnosis, (4) cancer stage, (5) intent to treat (6) and treatment options.

### Data Analysis

Stata statistical software version 14 was used for analysis. Socioeconomic status was generated as a single explanatory variable using factor analysis based on a set of variables namely: type of residence, house ownership, energy source; mode of transport, communication facilities, water source and type of toilet (flush toilet) because they were all indicators of socioeconomic profile. In factor analysis, first level explained largest proportion of total variance and assets that were more unequally distributed across the sample had higher weights. Those weights were used for each asset to generate factor scores. Higher score indicated higher wealth status and vice versa. Finally based on quintiles, the scores were converted into five ordered categories from highest (1st quintile) to lowest (5th quintile). Therefore, the new variable SES was categorized into those five categories namely highest, higher, high, middle and low.

We estimated correlation between QoL and various sociodemographic characteristics. Chi-square test was used to assess the association between quality of life and explanatory variables. An unadjusted logistic regression model was used to identify explanatory variables associated with quality of life. All significant explanatory variables (p<0.05) in the adjusted model were all fitted into multivariate logistic regression model using forward selection to determine factors independently associated with quality of life at p<0.05. The model was tested for sensitivity by the forward selection procedure (e.g., including and excluding specific variables) with robust standard errors.

## Results

### Sociodemographic characteristics of the study participants

A total of 398 participants were included in the analysis and the majority were females (64%). The highest proportions were in the middle age group of 45–54 years (N=136, 34%) with an average age of 43 years (standard deviation=12.46). Cervical cancer was the commonest malignancy (30%), seconded by Kaposi ‘sarcoma (24%). Other malignancies included the following; breast (11%), esophageal (4%), leukemia (4%) and non-Hodgkin's lymphoma (3%). The most common treatment modalities were; chemotherapy (99%), surgery (20%), radiation (0.26%), herbal remedies (12%), spiritual healing (24%). Most patients (74%) were being managed with curative intent. At least 18% had missed their clinical appointments largely due to high transport costs (73%), disease severity (29%) and long distances of travel (29%) as shown in table 1.

**Table 1:** Sociodemographic characteristics by facility

Patient Characteristics	QECH: N=205, n (%)	KCH: N=193, n (%)	Total: N=398, n (%)	P-value†
Gender				
Female	127 (61.95)	128(66.32)	255 (64.07)	
Male	78 (38.65)	65 (33.68)	143 (35.93)	0.364
Age (years)	43 ± 12.46	47 ± 12.90	45 ± 12.77	
18–24	15 (7.32)	5 (2.59)	20 (5.03)	
25–34	29 (14.15)	28 (14.51)	57 (14.32)	
35–44	69 (33.66)	53 (27.46)	122 (30.65)	
45–54	68 (33.17)	68 (35.23)	136 (34.17)	
55–64	24 (11.71)	39 (20.21)	63 (15.83)	0.035*
Marital status				
Never married	15 (7.32)	14 (7.25)	29 (7.29)	
Currently married	137 (66.83)	126 (65.28)	263 (66.08)	
Divorced	31 (15.12)	28 (14.51)	59 (14.82)	
Widow	22 (10.73)	25 (12.95)	47 (11.81)	0.924
Education status				
No education	35 (17.07)	39 (20.31)	74 (18.64)	
Primary	93 (45.37)	90 (46.88)	183 (46.10)	
Secondary	64 (31.22)	52 (27.08)	116 (29.22)	
Tertiary	13 (6.34)	11 (5.73)	24 (6.05)	0.741
Occupation status				
Not employed	102 (49.76)	78 (40.63)	180 (45.34)	
Formally employed	23 (11.22)	17 (8.85)	40 (10.08)	
Informally employed	52 (25.37)	79 (41.15)	131 (33.00)	
Student	5 (2.44)	4 (2.08)	9 (2.27)	
Retired	5 (2.44)	7 (3.65)	12 (3.02)	
Others	18 (8.78)	7 (3.65)	25 (6.30)	<0.013*
Area of residence				
Urban	92 (44.88)	62 (32.29)	154 (38.79)	
Rural	113 (55.12)	130 (67.71)	243 (61.21)	<0.01*
Socioeconomic status				
Highest	59 (28.78)	37 (19.27)	96 (24.18)	
Higher	35 (17.07)	34 (17.71)	69 (17.38)	
High	27 (13.17)	51 (26.56)	78 (19.68)	
Middle	65 (31.71)	55 (28.65)	12 (30.23)	
Low	19 (9.27)	15 (7.81)	34 (8.56)	<0.01*
Smoking history				
Never smoked	121 (83.05)	168 (87.05)	339 (85.18)	
Ever smoked	29 (14.15)	22 (11.40)	51 (12.81)	
Current smokers	5 (2.44)	3 (1.55)	8 (2.01)	0.569
Alcohol history				
Never alcohol	156 (76.10)	157 (81.35)	313 (78.64)	
Ever alcohol	43 (20.98)	31 (16.06)	74 (18.59)	
Current alcohol	6 (2.93)	5 (2.59)	11 (2.76)	0.432
Body mass index				
Underweight	32 (15.61)	23 (11.92)	55 (13.82)	
Normal weight	98 (47.80)	128 (66.32)	226 (56.78)	
Over weight	47 (22.93)	32 (16.58)	79 (19.85)	
Obesity	28 (13.66)	10 (5.18)	38 (9.55)	<0.001*
Cancer diagnosis				
Kaposi's sarcoma	46 (22.44)	51 (26.42)	97 (24.37)	
Cervical	52 (25.37)	69 (35.75)	121 (30.40)	
Breast	19 (9.27)	23 (11.92)	42 (10.55)	
Esophageal	12 (5.85)	5 (2.59)	17 (4.27)	
Leukemia	15 (7.32)	1 (0.52)	16 (4.02)	
Non-Hodgkin's lymphoma	7 (3.41)	4 (2.07)	11 (2.76)	0.003*
Cancer stage				
Localized	186 (90.73)	128 (66.32)	314 (78.89)	
Lymph node involvement	17 (8.29)	51 (26.42)	68 (17.09)	
Distant metastasis	2 (0.98)	14 (7.25)	16 (4.02)	<0.001*
Treatment modality				
Chemotherapy	204 (99.51)	180 (98.36)	384 (98.97)	0.262
Surgery	27 (13.17)	51 (27.87)	78 (20.10)	<0.001*
Radiation	-	1 (0.55)	1 (0.26)	0.289
Herbal remedies	5 (2.44)	42 (22.95)	47 (12.11)	<0.001*
Spiritual healing	12 (5.85)	83 (45.36)	95 (24.48)	<0.001*
Intention for treatment				
Cure	133 (77.78)	76 (67.26)	209 (73.59)	
Palliative	38 (22.22)	37 (32.74)	75 (26.41)	0.049*
Psychosocial experiences				
Clinical Appointment				
Missed clinical appointment	39 (19.02)	34 (17.62)	73 (18.34)	0.717
Reasons of missed appointment				
Lack of transport	32 (82.05)	22(64.71)	54 (73.97)	0.092
Disease severity	15 (44.12)	6 (15.38)	21 (28.77)	0.007*
Long distance	18 (46.15)	3 (9.09)	21 (29.17)	<0.001*

* Denotes statistical significance at p-value <0.05 (p-values from Pearson's Chi-square correlation) and confidence interval of 95%.

† Denote the p-values comparing two facilities under study. QECH= Queen Elizabeth Central Hospital, KCH= Kamuzu Central Hospital.

### Psychosocial experiences of the study participants

Patients reported the following psychosocial challenges; pill burden (58%), loss of earning (78%), fear of death (18%). Participants suggested the following responses to solve their psychosocial challenges; reminders using Short Messaging Service (SMS) (52%), formulation of patient support groups (70%), decentralized cancer care (77%) regular health talks (62%) and home visits (79%).

### Quality of life experiences of the study participants

In five dimensions of quality-of-life scale, majority (75%) had no mobility problems while 24% of the participants had some mobility challenges. At least 91% had no problems with self-care and that about 8% of the participants had problems with daily usual activity. Pain was the most prevalent problem experienced by cancer patients as 44% had some pain while 9% had extreme pain. We observed that 27% of the study participants had some anxious perception about their disease situation while 13% had extreme anxiety problems. At most 23% had worst imaginable health status on the subjective visual analogues scale as presented in table 3.

**Table 3:** Factors associated with quality of life among cancer patients

Factor	Unadjusted OR (95% CI)	P-value	Adjusted OR (95% CI)	p-value
Gender				
Female	1.00		1.00	
Male	0.78 (0.49–1.24)	0.297	0.52 (0.23–1.18)	0.121
Age				
18–24	1.00		1.00	
25–34	1.00 (0.35 –2.84)	1.00	0.67 (0.13 – 3.39)	0.631
35–44	1.98 (0.74 –5.31)	0.177	1.42 (0.29 – 6.79)	0.661
55–64	1.85 (0.69 –4.90)	0.217	0.89 (0.18 – 4.51)	0.894
55–64	1.83 (0.63 – 5.30)	0.263	0.86 (0.16 –4.78)	0.865
Facility				
KCH	1.00		1.00	
QECH	0.41 (0.26 – 0.65)	<0.001*	0.29 (0.17 – 0.54)	<0.001
Education status				
No education	1.00		1.00	
Primary	1.19 (0.64 – 2.22)	0.574	1.96 (0.91 – 4.26)	0.087
Secondary	0.71 (0.37 – 1.37)	0.31	1.67 (0.66 – 4.22)	0.277
Tertiary	0.53 (0.20 – 1.38)	0.193	0.93 (0.24 – 3.67)	0.919
Occupation status				
No employment	1:00		1.00	
Formal employment	0.65 (0.31 –1.34)	0.244	0.97 (0.37 – 2.53)	0.944
Informal employment	0.74 (0.45 – 1.23)	0.248	0.61 (0.33 – 1.13)	0.115
Socioeconomic status				
Highest	1.00		1.00	
Higher	0.91 (0.48 –1.74)	0.774	0.72 (0.32–1.63)	0.434
High	1.49 (0.77 – 2.92)	0.234	1.09 (0.44 – 2.73)	0.848
Middle	2.33 (1.25 – 4.34)	0.008*	2.46 (0.99 – 6.10)	0.051
Low	2.32 (0.87– 5.65)	0.093	3.39 (0.93 – 12.36)	0.064
Body mass index				
underweight	1:00		1.00	
Normal weight	0.26	0.007*	0.25 (0.08 – 0.74)	0.012*
Over weight	0.15	<0.001*	0.17 (0.05 – 1.23)	0.064
obesity	0.22 (0.85 –4.93)	0.009*	0.29 (0.07 –1.10)	0.068
Alcohol history				
Never alcohol	1.00		1.00	
Ever alcohol	1.56 (0.85 –2.86)	0.154	2.36 (1.02–5.44)	0.045*
Current drinker	2.00 (0.42 – 9.44)	0.381	3.33 (0.47–23.84)	0.23
Disease comorbidity				
No comorbidity	1.00		1.00	
1–2 comorbid conditions	0.72 (0.45 – 1.16	0.177	1.06 (0.58 – 1.92)	0.849
3 or more comorbid conditions	2.22 (0.81 – 6.13)	0.123	3.78 (1.08 – 13.12)	0.037*
Cancer diagnosis				
Kaposi sarcoma	1.00		1.00	
Cervical	0.99 (0.55–1.81)	0.989	0.62 (0.25–1.54)	0.305
Esophageal	1.88 (0.50–7.07)	0.35	1.69 (0.34 – 8.39)	0.518
Non-Hodgkin's lymphoma	0.67 (0.15 – 3.01)	0.603	0.60 (0.09 – 4.10)	0.604
Leukemia	0.46 (0.15 – 1.40)	0.17	0.96 (0.26 – 3.61)	0.954
Breast	1.06 (0.47–2.43)	0.886	1.20 (0.40 – 3.62)	0.742

* Denotes statistical significance at p-value <0.05, CI= Confidence Interval, AOR = Adjusted Odds Ratio

### Factors ass ociated with quality of life among cancer patients

In adjusted regression model, being treated at QECH was associated with improved quality of life, 0.29 (95% CI: 0.17–0.54, P<0.001) as well as normal weight, 0.25 (95% CI: 0.08–0.74, P=0.012). On the other hand, patients with history of ever taken alcohol, 2.36 (95% CI: 1.02–5.44, P=0.045) and having 3 or more comorbid conditions, 3.78 (95% CI: 1.08–13.12, p=0.037) were associated with poor quality of life.

## Discussion

Many cancer patients experience both physiological and psychosocial challenges. In this study, we aimed at estimating quality of life with focus on psychosocial well-being among cancer patients in two referral hospitals in Malawi. Cervical cancer remained the leading cause of morbidity among Malawi cancer patients and this was consistent with country GLOBOCAN recent report for 2020 7. Most participants (74%) were managed with intention to cure the disease. This implied that they were in early stages of their cancer disease process. This was an important finding considering that early diagnosis is associated with higher remission rate given availability of all treatment modalities. However, at the time of the study, the country had not commissioned any radiotherapy services^22^. Yet radiation medicine technology is a critical and indispensable component of comprehensive cancer treatment and care with approximately above 50% of the all cancers requiring radiation for diagnostic, treatment and palliative care services among cancer patients 23–25.

In terms of psychosocial experiences, higher proportions of participants had lost earnings and failed to provide for their dependents and was consistent with findings from other studies 18, 26, 27. Cancer diagnosis and treatment often overshadow the impact of financial burden on QoL due to inability to work and out-of-pocket costs expenditure on livelihood, transport and medical bills 28–30. Marital problems were also common in this present study as well as disfigurement, sexual dysfunction and loss of energy which could impact negatively on self-esteem and general coping mechanisms against the disease 31. Other participants experienced fear of death, a similar experience reported by other studies as well 27, 31, 32.

We also found that significant proportions of the participants complained of pill burden, side effects which had potential of reducing drug compliance. 31. Low compliance is a major barrier to good health outcomes 33. On other hand, participants had proposed some solutions including use of SMS as a temporal reminder to curb low compliance and in other studies this has been successful (84% versus 77%) in achieving good outcomes as clients felt cared 33, 34. However, accessibility to mobile phones could be a challenge among our participants because of low subscription rate of mobile phones in Malawi at 48% in 2019 according to world bank report 35.

Use of support groups has also been proposed by participants in this present study. These are meetings for people with cancer and anyone touched by the disease^36^. Patients realized that joining support groups with others who have similar cancer experiences could improve their quality of life and survival 36. Support groups can help patients to feel better, give them opportunity to talk about their feelings, help deal with problems and coping to treatment side effects. They are many types of support groups such as: online, peer-to-peer, groups for cancers in general, groups for particular cancer type, groups for patients / or families and caregivers 36, 37. The need for support groups for cancer have also been recommended not only by patients themselves but also with health professionals elsewhere 30. Health education either through clinic-based or home visits have also been proposed as options to improve psychosocial challenges among cancer patients in this current study. In fact, health education as an intervention administered at the clinic has been effective towards improving pain, distress, anxiety, depression, quality of life and performance among cancer patients 38. Participants also proposed that decentralized cancer services to district hospitals would be ideal to cut short of transport costs. Elsewhere, longer distances of travel in seeking health services have been associated with increased burden on value for time, cost implications as well as discomfort to patients 39. Transport support in form of subsidies by government could go a long way in reducing travel cost 18.

On QoL scale, despite having better mobility (75%), self-care (91%) and usual activity (70%) scores on QoL scale, most patients experienced some pain and anxiety and these results were similar to the findings from other non-cancer patients with chronic diseases on palliative care in Malawi 17, 18. Between the two facilities under study, patients from QECH had improved quality of life than KCH. The reasons for these differences remained unknown however, QECH has an older and established cancer unit compared to KCH. Above all, QECH is the main teaching hospital which would benefit from students' allocation in wards from College of Medicine (Kamuzu University of Health Sciences), the only medical institution in the country at the time of the conduct of the study. We also observed that normal weight was associated with improved quality of life and the findings were consistent with other studies 40–43. Having multiple (3 or more) chronic disease comorbidities was significantly associated with poor quality of life. These results were consistent with findings from other studies elsewhere 21,28, 44. A positive history of taking alcohol in the past was also associated with poor quality of life. Despite having different cancers, the study has reported no differences in QoL between cancers, sex distribution and age. The study also did not report any association between quality of life and socioeconomic status, occupation and education among cancer patients. Therefore, in the present study, the determinants of QoL included: facility type, multiple disease comorbidities, ever taken alcohol history, normal weight and overweight. This study was limited by its design as it failed to establish causal relationship between the quality of life and exposure variables. In addition, it failed to capture common side-effects experienced by participants. The study only focused on the QoL for all cancer patients without specifying cancer type yet there could be important variations in QoL between cancers. Furthermore, although the study was conducted on two major referral facilities, the findings may not be representative of all cancer patients' experiences in Malawi however, the findings could provide useful information for further studies. Apart from being conducted on two main tertiary hospitals in Malawi, the other strength derived from the study was its utilization of EQ-5D-3L tool for measuring quality of life which was validated in Malawi. In conclusion, most participants in this study had complained of loss of earning, pain, marital strife, sexual dysfunction, as common psychosocial challenges experienced by cancer patients which affected their quality of life. History of ever taken alcohol and having 3 or more comorbidities were associated with poor quality of life. There is need to integrate mandatory and comprehensive psychosocial education, psychosocial support and psychotherapy for cancer patients and their families to improve their quality of life and outcomes.

## Figures and Tables

**Figure 1:**
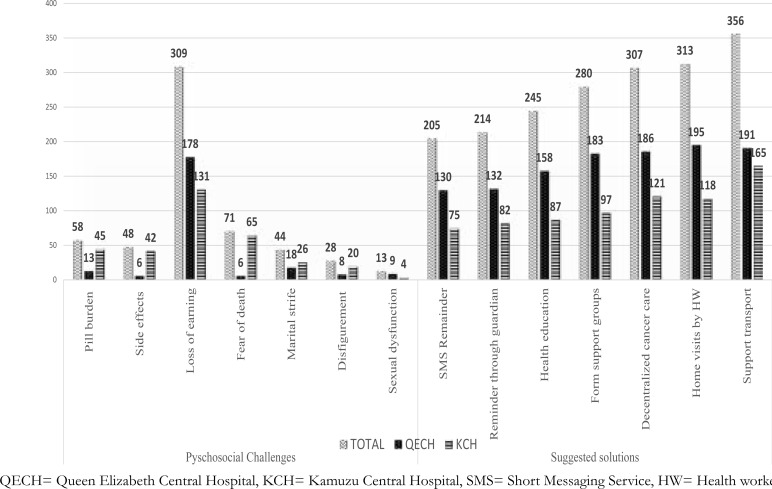
Psychosocial experiences of study participants

**Table 2:** Quality of life distribution of the study participants

Quality of life characteristics	QECH: N=202 n (%)	KCH: N=180 n (%)	TOTAL: N=382 n (%)	P-value
Mobility				
No problem	163 (80.69)	124 (68.89)	287 (75.13)	
Some problem	39 (19.31)	51 (28.33)	90 (23.56)	
Extreme problem	-	5 (2.78)	5 (1.31)	<0.005*
Self-care				
No problem	191 (94.55)	158 (87.78)	349 (91.36)	
Some problem	9 (4.46)	17 (9.44)	26 (6.81)	
Extreme problem	2 (0.99)	5 (2.78)	7 (1.83)	0.06
Usual activity				
No problem	135 (66.83)	134 (74.44)	269 (70.49)	
Some problem	52 (25.74)	34 (18.89)	86 (22.51)	
Extreme problem	15 (7.43)	12 (6.67)	27 (7.67)	0.242
Pain				
No problem	112 (55.45)	66 (36.67)	178 (46.60)	
Some problem	86 (42.57)	83 (46.11)	169 (44.24)	
Extreme problem	4 (1.98)	31 (17.22)	35 (9.16)	<0.001*
Anxiety/ depression				
No problem	160 (79.21)	68 (37.78)	228 (59.69)	
Some problem	36 (17.82)	67 (37.22)	103 (26.96)	
Extreme problem	6 (2.97)	45 (25.00)	51 (13.35)	<0.001*
Visual Analogue scale (VAS)				
Worst imaginable health	-	84 (46.67)	84 (21.99)	
status				
Better imaginable health status	19 (9.41)	67 (37.22)	86 (22.51)	
Best imaginable health status	183 (90.59)	29 (16.11)	212 (55.50)	<0.001*

* Denotes statistical significance at p-value <0.05 (p-values from Pearson's Chi-square correlation) and confidence interval of 95%. QECH= Queen Elizabeth Central Hospital, KCH= Kamuzu Central Hospital.

## Data Availability

All relevant data are available within the paper. Individual level data are not freely available for ethical reasons as public availability would compromise patient confidentiality. Additional data requests can be sent to the author at jonchiwanda@gmail.com.

## What is already known on this topic

- Cancer burden on the increase worldwide.
- Burden disproportionately affected Low- and Middle-Income Countries (LMICs) which contributed 70% of cancer deaths in 2020.
- Cancer patients experience various psychosocial challenges.

## What this study adds

- Cancer patients experience marital strife, loss of earning, pain, sexual dysfunction and fear of death.
- QECH was associated with an improved QoL for cancer patients.
- Having 3 or more comorbidities was associated with poor QoL
